# Chromosome-level genome assembly of the fully mycoheterotrophic orchid *Gastrodia elata*

**DOI:** 10.1093/g3journal/jkab433

**Published:** 2022-01-19

**Authors:** Eun-Kyung Bae, Chanhoon An, Min-Jeong Kang, Sang-A Lee, Seung Jae Lee, Ki-Tae Kim, Eung-Jun Park

**Affiliations:** 1 Forest Microbiology Division, National Institute of Forest Science, Suwon 16631, Korea; 2 Division of Biotechnology, College of Life Sciences and Biotechnology, Korea University, Seoul 02841, Korea; 3 Department of Agricultural Life Science, Sunchon National University, Suncheon 57922, Korea

**Keywords:** *Gastrodia elata*, genome assembly, mycoheterotrophic, Orchidaceae, pseudochromosome

## Abstract

*Gastrodia elata*, an obligate mycoheterotrophic orchid, requires complete carbon and mineral nutrient supplementation from mycorrhizal fungi during its entire life cycle. Although full mycoheterotrophy occurs most often in the Orchidaceae family, no chromosome-level reference genome from this group has been assembled to date. Here, we report a high-quality chromosome-level genome assembly of *G. elata*, using Illumina and PacBio sequencing methods with Hi-C technique. The assembled genome size was found to be 1045 Mb, with an N50 of 50.6 Mb and 488 scaffolds. A total of 935 complete (64.9%) matches to the 1440 embryophyte Benchmarking Universal Single-Copy Orthologs were identified in this genome assembly. Hi-C scaffolding of the assembled genome resulted in 18 pseudochromosomes, 1008 Mb in size and containing 96.5% of the scaffolds. A total of 18,844 protein-coding sequences (CDSs) were predicted in the *G. elata* genome, of which 15,619 CDSs (82.89%) were functionally annotated. In addition, 74.92% of the assembled genome was found to be composed of transposable elements. Phylogenetic analysis indicated a significant contraction of genes involved in various biosynthetic processes and cellular components and an expansion of genes for novel metabolic processes and mycorrhizal association. This result suggests an evolutionary adaptation of *G. elata* to a mycoheterotrophic lifestyle. In summary, the genomic resources generated in this study will provide a valuable reference genome for investigating the molecular mechanisms of *G. elata* biological functions. Furthermore, the complete *G. elata* genome will greatly improve our understanding of the genetics of Orchidaceae and its mycoheterotrophic evolution.

## Introduction

Mycoheterotrophy represents one extreme end in the mutualism-parasitism continuum of mycorrhizal symbiosis (Leake 1994), upon which the largest number of vascular plant species depend (Leake 2005). In total, more than 450 vascular plant species maintain a fully mycoheterotrophic lifestyle throughout their entire lives without producing green leaves (Merckx and Freudenstein 2010). Full mycoheterotrophy occurs in a wide phylogenetic range of plant species, especially culminating in the Orchidaceae, the most widely distributed plant family on Earth (Leake 1994). This family contains the largest number of fully mycoheterotrophic species (at least 210) (Merckx and Freudenstein 2010).

*Gastrodia elata* Blume is a fully mycoheterotrophic orchid that is symbiotically associated with at least two fungal partners: a broad range of *Mycena* spp. are required for seed germination (Xu and Guo 1989; Shunxing and Qiuying 2001; Park and Lee 2013) and *Armillaria mellea* is essential for plant growth (Zhang and Li 1980). Such mycorrhizal community changes during ontogenetic development have been shown in other species. For example, the fungi that associate with seeds of several *Pyrola* (Ericaceae family) species differ from those coupled with adult plants (Hashimoto *et al.* 2012; Hynson *et al.* 2013; Johansson *et al.* 2017; Jacquemyn *et al.* 2018). This indicates that some plants may serially associate with different fungal partners rather than choosing a single best partner. Similarly, the mycorrhizal communities associated with protocorms and adult plants of the orchid *Liparis loeselii* are also diverse, varying among the different life cycle stages (Waud *et al.* 2017).

In the last decade, a number of genomic resources have been developed to study the mycoheterotrophic adaptation of *G. elata*, including transcriptomes (Tsai *et al.* 2016; Zeng *et al.* 2017; Wang *et al.* 2020), proteomic data (Zeng *et al.* 2018), and a draft genome assembly (Yuan *et al.* 2018). The previous *G. elata* genome assembly, determined with the Illumina HiSeq 2500 platform, was highly fragmented (Yuan *et al.* 2018). In particular, the low contiguity of this genome assembly has limited its application for further research on the genomic evolution of *G. elata*. Moreover, no chromosome-level genome has ever been assembled for a member of the obligate mycoheterotrophic Orchidaceae family. Therefore, an accurate genome assembly of *G. elata* is essential for both basic and applied research, which will improve our understanding of genome evolution in the Orchidaceae family and accelerate the genetic improvement for *G. elata* cultivation in the commercial field for food and medicine.

Here, we present a vastly improved de novo assembly and annotation of the *G. elata* reference genome using these new sequencing technologies, including single-molecule real-time (SMRT) sequencing from Pacific Biosciences (PacBio) and chromosome conformation capture (Hi-C) (Wingett *et al.* 2015; Korlach *et al.* 2017; Pennisi 2017). Notably, this new assembly greatly improves genome completeness and contiguity over the previous version of the reference genome. Last, comparative analysis with other orchid species revealed the emergence of evolutionary novelties and functional diversification of *G. elata*, leading to the development of the unique mycoheterotrophic lifestyle.

## Materials and methods

### Sample collection and DNA sequencing

**Figure 1:**
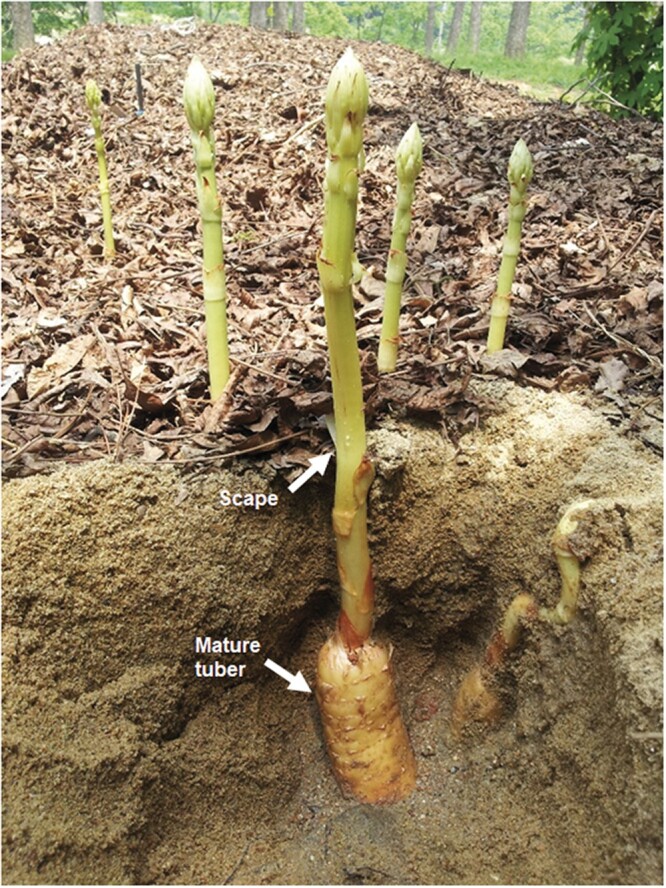
Photograph of *Gastrodia elata*. The white arrows indicate the mature tuber and scape.

Experimental sample of *G. elata* was collected from Muju (35˚51’N 127˚39’E; 510-m altitude) in Jeollabuk-do Province, which is located in southern Korea (Figure 1). High-molecular-weight genomic DNA (gDNA) was isolated from a single genotype of *G. elata* scape, using the modified cetyltrimethylammonium bromide (CTAB) method (Inglis *et al.* 2018), and the high-quality gDNA was purified using the DNeasy Plant Mini Kit (Qiagen, Hilden, Germany) after RNase A treatment. The quantity of the extracted DNA was then determined using a 2100 Bioanalyzer (Agilent Technologies, Santa Clara, CA, USA).

To perform the genomic survey, an Illumina paired-ended DNA library, with an insert size of 550 bp, was prepared according to the Illumina TruSeq DNA PCR-Free Library Prep protocol (Illumina, San Diego, CA, USA). The Agilent 2100 Bioanalyzer High Sensitivity Kit was used to check for quality, and the library was sequenced on the Illumina NovaSeq 6000 platform, using a 150-bp paired-end strategy.

For long-read sequencing, 25 SMRTbell 20 kb DNA libraries were constructed using the following steps, according to the PacBio standard protocol: (1) gDNA shearing using the Covaris g-TUBE (Covaris Inc., Woburn, MA, USA); (2) DNA damage repair; (3) blunt-end ligation with hairpin adapters from the SMRTbell Template Prep Kit 1.0 (PacBio, Menlo Park, CA, USA); (4) 20 kb size-selection using the BluePippin Size Selection System (Sage Science, Beverly, MA, USA); and (5) binding to polymerase using the MagBead Kit (Pacific Biosciences, Menlo Park, CA, USA). Subsequently, SMRT long-read sequencing was performed on a PacBio Sequel platform with the Sequel Sequencing Kit 2.1.

A Dovetail Hi-C library was constructed from a scape tissue according to the manufacturer’s instructions (Dovetail Hi-C Library kit), and sequenced with the Illumina NovaSeq 6000 platform, according to published protocols (Lieberman-Aiden *et al.* 2009). A scape tissue was cross-linked with PBS/formaldehyde, and then chromatin was prepared with SDS and wash buffer. After normalizing the chromatin plant sample, 800 ng of chromatin was used to make the library. Chromatin was captured by chromatin capture beads and then digested with restriction enzyme. Its end was filled in with biotin and ligated to form Intra-aggregated DNA. After cross-link reversal, 200 ng of DNA was sheared using the Covaris system. Sheared DNA fragments were end-repaired and ligated with Illumina adapter. Ligated DNA was purified using Streptavidin Magnetic Beads. Purified DNA was amplified by PCR to enrich fragments. The quality of the amplified libraries was verified by capillary electrophoresis (Bioanalyzer, Agilent). Sequencing is performed using an Illumina NovaSeq 6000 system following provided protocols for 2 × 150 sequencing. In summary, Hi-C fragment libraries were prepared according to the “Proximo Hi-C protocol” with *Dpn*II digest, and the resulting libraries were sequenced using a 150-bp paired-end strategy.

### Genome assembly

Raw Illumina paired-end sequencing reads were filtered using the FASTP v.0.12.6 preprocessor (set to default parameters) to remove low-quality reads, adapters, and reads containing poly-*N* (Chen *et al.* 2018). Trimmed Illumina sequencing reads were then used to calculate the percentage of heterozygosity in the genome. For this analysis, Jellyfish v.2.2.10 was first used to compute the histogram of 19 k-mer frequencies (Marcais and Kingsford 2011), and genome heterozygosity was then calculated by the GenomeScope v.2.0 online platform, using the final k-mer count histogram (Vurture *et al.* 2017).

To perform de novo genome assembly, we used the FALCON-Unzip assembler v0.4 (Chin *et al.* 2016), with length cutoff parameters (length cutoff = 13 kb, length cutoff pr = 10 kb) and filtered subreads from SMRT Link v.5.0.0 (minimum subread length = 50 bp). To improve accuracy of the assembly, the FALCON-Unzip assembler was polished with the Arrow algorithm, using the PacBio unaligned BAM files as raw data. Then, the error correction was performed with alignment from the short-read using Pilon v.1.23 (Walker *et al.* 2014).

The falcon-unzip assembly and Dovetail Hi-C reads were then used as input data for HiRise, a software pipeline designed specifically for utilizing proximity ligation data to scaffold genome assemblies (Putnam *et al.* 2016). Hi-C library sequences were aligned to the draft input assembly using a SNAP read mapper (Zaharia *et al.* 2011). The separations of Hi-C read pairs mapped within draft scaffolds were analyzed by HiRise to produce a likelihood model for the genomic distance between read pairs. This model was then used to identify and break putative misjoins, score prospective joins, and make joins above a threshold. Finally, organelle genomes were filtered out from public organelle sequences in NCBI using BLAST v.2.4.0 (Altschul *et al.* 1990), and completeness of the genome assembly was assessed using Benchmarking Universal Single-Copy Orthologs (BUSCO) v.3.0.1 with default parameters and the embryophata dataset (Simao *et al.* 2015).

### Transcriptome sequencing

Tissue samples were collected through the 12 development stages of *G. elata*. The collected samples were immediately frozen in liquid nitrogen and stored at −80°C until RNA extraction. Total RNA was extracted from each sample with TRIzol reagent (Invitrogen, Waltham, MA, USA). RNA quality and quantity were checked using the Bioanalyzer 2100 system (Agilent Technologies, Santa Clara, CA, USA). The full-length cDNA library was generated using 1 μg of equally mixed RNA from the 12 different tissues and the Clontech SMARTer PCR cDNA Synthesis Kit according to the Isoform Sequencing protocol (PacBio, Menlo Park, CA, USA). PCR optimization was carried out on the full-length cDNA using the PrimeSTAR GXL DNA Polymerase (Clontech, Mountain View, CA, USA) and 12 cycles were sufficient to generate the material required for SMRTbell library preparation. Each cDNA sample was bead cleaned with AMPure PB beads post PCR in preparation for SMRTbell library construction. The sequencing primer from the SMRTbell Template Prep Kit 1.0-SPv3 was annealed to the adapter sequence of the libraries. Each library was bound to the sequencing polymerase with the Sequel Binding Kit v2.1 and the complex formed was then purified using AMPure Purification (Clontech, Mountain View, CA, USA). The libraries were sequenced using 2 SMRTcells v2.0 per library on the Sequel sequencing platform. All libraries had 600-min movies and 240 min of pre-extension time. The full-length isoform sequence was constructed using SMRTLink v.5.1 (Pacific Bioscience, CA, USA) through several steps. First, the qualified sequence was classified based on detection of primers and polyA tail. Then, the isoform sequence was generated with isoform-level clustering that was categorized as high-quality based on over 99% estimated accuracy.

### Genome annotation

The *G. elata* genome was annotated using custom repeat library protocols, ab initio gene prediction, homology search, and full-length transcript evidences. A de novo repeat library was constructed using RepeatModeler v.1.0.3 (Price *et al.* 2005), including RECON v.1.08 (Bao and Eddy 2002) and RepeatScout v.1.0.5 (Price *et al.* 2005) with default parameters. Tandem Repeats Finder v.4.09 (Benson 1999) was used to predict consensus sequences, classification information for each repeat, and tandem repeats, including simple repeats, satellites, and low complexity repeats (Benson 1999). To identify highly accurate long terminal repeat retrotransposons (LTR-RTs), we constructed an LTR library with LTR_retriever v.2.9.0 (Ou and Jiang 2018), using combined raw LTR data from LTRharvest v.1.6.1 (Ellinghaus *et al.* 2008) and LTR_FINDER v.1.0.7 (Xu and Wang 2007). Repetitive elements in the de novo repeat library were identified using RepeatMasker v.4.0.9, and the quality of repetitive elements was assessed using LTR Assembly Index (LAI) program (Ou *et al.* 2018). Kimura distances (Kimura 1980) for all transposable element (TE) copies from each family found in the library were calculated to estimate the age of TEs.

Genome annotation was performed with MAKER v.2.31.8 (Holt and Yandell 2011), using three rounds of reiterative training (Holt and Yandell 2011). Subsequently, *ab initio* gene prediction was performed with SNAP v.2006-07-32 (Korf 2004) and Augustus v.3.3.3 (Stanke *et al.* 2006). MAKER was initially run in est2genome mode based on full-length transcripts from Iso-Seq data. In addition, evidence for protein-coding genes was obtained from the genomes of three orchid plants: *Apostasiashenzhenica* (GCA_002786265.1) (Zhang *et al.* 2017), *Dendrobiumcatenatum* (GCA_001605985.2) (Zhang *et al.* 2016), and *Phalaenopsisequestris* (GCA_001263595.1) (Cai *et al.* 2015). Exonerate v2.4.0 (Slater and Birney 2005), which provides integrated information for the SNAP program, was used to polish MAKER alignments. Other noncoding RNAs were identified using the Barrnap v0.9 (https://vicbioinformatics.com/software.barrnap.shtml). The putative tRNA genes were identified using tRNAscan-SE v2.0.5 (Chan and Lowe 2019). To select the best-supported gene models, we used a quality metric called annotation edit distance (AED), developed by the Sequence Ontology project (Eilbeck *et al.* 2009). More than 90% of our annotations had an AED score less than 0.5 (Campbell *et al.* 2014).

For functional annotation, predicted proteins were aligned to the National Center for Biotechnology Information (NCBI) nonredundant protein databases (Marchler-Bauer *et al.* 2011), using BLAST v.2.4.0 (Altschul *et al.* 1990) with a maximum *e*-value cutoff of 1e-5. Protein signatures were annotated using InterProScan v.5.44.79 (Jones *et al.* 2014) for further BLAST2GO v.5.2.5 (Götz *et al.* 2008) based gene ontology (GO) analysis (Dimmer *et al.* 2012). Predicted proteins were also searched against the Kyoto Encyclopedia of Genes and Genomes (KEGG) database to retrieve KEGG-relevant functional annotations.

### Gene family identification and phylogenetic analysis

Orthologous gene clusters were classified within the genomes of 15 plant species, including *G. elata* (Supplementary Table S1), using OrthoMCL v2.0 (OrthoMCL-DB: Ortholog Groups of Protein Sequences) (Li *et al.* 2003). We then extracted the longest protein sequence isoforms from the gene predictions of each plant species with default parameters to construct a phylogenetic tree, using Orthofinder v2.4.0 (Emms and Kelly 2019) with an *e*-value cutoff 1e-5 and all-to-all BLASTP analysis of the 15 plant species. MAFFT v.6.861b (Katoh *et al.* 2009) was used to align each gene family, and the phylogenetic tree was inferred with FastTree v.2.1.10 (Price *et al.* 2010), with divergence time calibration performed using both PATHd8 (Britton *et al.* 2007) and TimeTree (Kumar *et al.* 2017). Last, CAFE v.4.2.1 (Han *et al.* 2013) was used to predict the likelihood of gene family expansion and contraction with *P <* 0.01 and automatic searching for the λ value.

## Results and discussion

### Genome assembly

**Figure 2:**
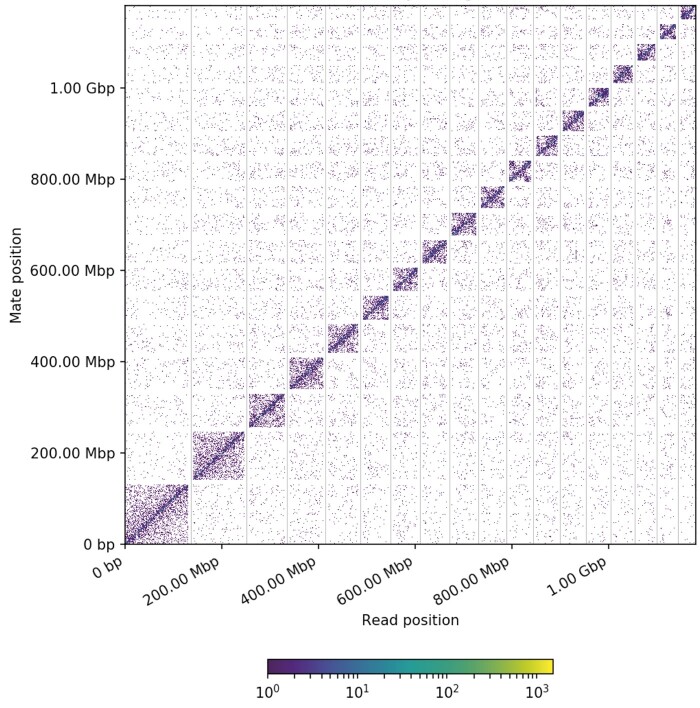
Genome-wide Hi-C interaction heatmap of *G. elata*. The 18 assembled scaffolds are ordered by length. The *x*- and *y*-axes provide the mapping positions for the first and second reads in each read pair, respectively, grouped into bins. The color of each square indicates the number of read pairs within that bin. Gray vertical and white horizontal lines have been added to indicate the borders between scaffolds. The off-diagonal pattern in the pseudochromosome 10 and 11 may reflect the Rabl configuration of chromatins (Tiang *et al.* 2012).

Using Illumina paired-ended sequencing, we first obtained 132.1 Gb of clean data after filtering out adapter sequences and low-quality reads. Prior to genome assembly, size of the *G. elata* genome was estimated from Illumina sequencing by GenomeScope, which predicted genome size of 1.023 Gbp, with heterozygosity of 0.06% (Supplementary Figure S1). We also performed long-read sequencing of the *G. elata* genome on the PacBio Sequel platform and obtained 11,449,345 PacBio long reads from 25 SMRT cells, representing a sequencing depth of 84.6X (Supplementary Table S2). FACON-Unzip was used to perform de novo assembly, and after the error correction step, we obtained a de novo assembly of 1.049 Gb, with a contig N50 of 9.18 Mb (Table 1), which is in broad agreement with the estimated genome size (1.023 Gb). Hi-C fragment library sequencing produced 121.6 Gb of clean data after filtering (Table 1). By mapping Hi-C sequencing data onto the genome assembly, we generated 32.33 Gb (52.6X coverage) of high-quality, validated Hi-C data to assemble contigs at the chromosome level (Supplementary Table S3). A total of 488 assembled contigs were anchored onto 18 pseudochromosomes that ranged from 32.1 to 130.6 Mb in length and contained 96.4% of the genome sequences (Figure 2; Supplementary Table S3). This chromosome number agrees with the previous karyotyping result of *G. elata* (Zhou *et al.* 2018). The pseudochromosome 10 and 11 showed an off-diagonal pattern, and the Rabl configuration of chromatids might cause it. The Rabl configuration is a description of interphase chromosome arrangement in which telomeres and centromeres are located at opposite sides of the nucleus (Tiang *et al.* 2012). For validation, the Illumina reads were aligned to the genome, and the percentage of proper pairs aligned was 96.11%.

**Table 1:** Assembly statistics of the *G. elata* genome

	FALCON-Unzip	HiRise	Final
Number of contigs (scaffolds)	654	514	488
Total size of contigs (scaffolds)	1,048,552,296	1,046,143,939	1,044,982,141
Longest contig (scaffold)	25,936,340	130,552,502	130,552,502
Number of contigs (scaffold) >1M nt	141	18	18
Number of contigs (scaffold) >10M nt	28	18	18
N50 contig (scaffold) length	9,175,439	50,595,616	50,595,616
L50 contig (scaffold) count	33	7	7
GC content (%)	34.27	34.27	34.27

FALCON-Unzip: Assembly result using PacBio data.

HiRise: Scaffolding result using FALCON-Unzip data.

Final: Organelle (plastid) genome removed from HiRise result.

Compared with the previous version of draft *G. elata* genome (Yuan *et al.* 2018), our genome assembly is greatly improved in terms of the number of scaffolds (488 *vs* 3779) and the length of scaffold N50 (50.6 *vs* 4.9 Mb). Among the Orchidaceae, our genome assembly is the first chromosome-level genome assembly of an obligate mycoheterotrophic orchid *G. elata*, although another chromosome-level reference genome is available for *P. aphrodite*, which is an epiphytic orchid (Chao *et al.* 2018). We further used BUSCO to assess the completeness of our genome assembly, based on the embryophyta_odb9 database (Table 2). We found that only 935 (64.9%) of the 1440 highly conserved orthologs are present as complete genes in the *G. elata* genome, indicating that 451 (31.3%) genes are missing from *G. elata*, which is consistent with results from the previous genome assembly (Yuan *et al.* 2018). The gene loss events are frequently observed in plastid genome of mycoheterotrophic orchids (Barrett and Davis 2012; Logacheva *et al.* 2014; Petersen *et al.* 2018; Kim *et al.* 2019), but only a few cases are reported in nuclear genome (Yuan *et al.* 2018; Jakalski *et al.* 2020). The extensive gene loss in nuclear genome could also be related to mycoheterotrophic lifestyle and may be associated with the large abundance of repetitive elements in *G. elata*.

**Table 2:** Statistics for genome assessment using BUSCO (embryophyta)

	No. of BUSCOs	Percentage of BUSCOs
Complete	935	64.9
Complete and single-copy	912	63.3
Complete and duplicated	23	1.6
Fragmented	54	3.8
Missing	451	31.3

### Genomic features and repetitive elements

**Figure 3:**
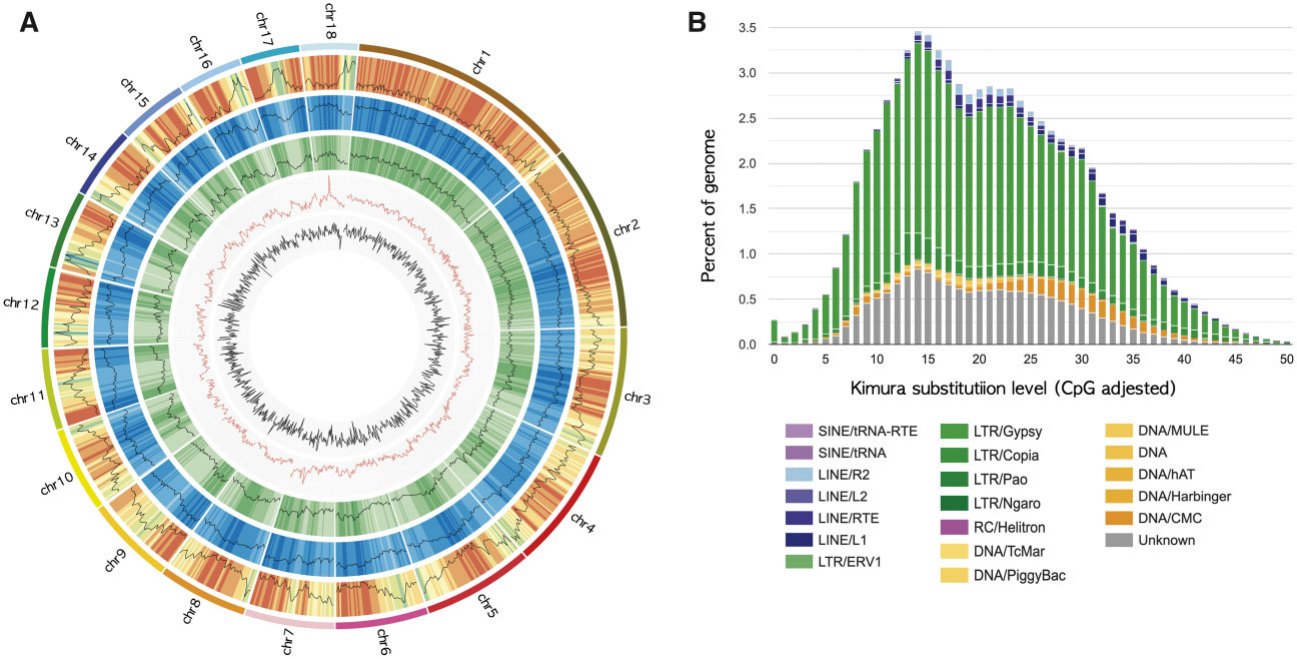
(A) Genome overview of the *G. elata* genome. The pseudochromosomes are in order from longest to shortest in a clockwise manner. The features are arranged in the order of gene density, repeat density, LTR/Gypsy, GC content, and GC skew from outside to inside in 1 Mb intervals across the 18 chromosomes. (B) Kimura distance-based copy divergence analysis of TEs in the *G. elata* genome. The graph represents the percentage of the genome represented by each repeat type on the *y*-axis to their corresponding Kimura substitution level (CpG adjusted) illustrated on the *x*-axis (*K*-value from 0 to 50). The color chart below the *x*-axis indicates the repeat types.

The gene density of orchid genomes, such as *P. aphrodite* (28.2 genes per Mb) and *P. equestris* (27.1 genes per Mb), is known to be lower than that of *Arabidopsis thaliana* (Cai *et al.* 2015; Chao *et al.* 2018), which is approximately 204.0 genes per Mb (calculated based on The *Arabidopsis* Genome Initiative 2000). Here, we found that the average gene density of the *G. elata* genome is 17.9 genes per Mb, with minimum and maximum densities on the first (Scx7bQ7_8: 10.8 genes per Mb) and 13th (Scx7bQ7_13: 22.5 genes per Mb) chromosomes, respectively (Figure 3A; Table 3). This is even lower than what has been detected in other orchid species. In contrast, the average repeat density was found to be 1406 repeats per Mb, and unlike genes, these are evenly distributed throughout the genome (Figure 3A). Retrotransposable elements, in particular, known to be the dominant form of repeats in angiosperm genomes (Oliver *et al.* 2013), constitute 74.92% (796.7 Mb) of the *G. elata* genome. This repetitive element content is higher than what has been found in any other orchid species, such as *A. shenzhenica* (47%), *P. equestris* (63.48%), and *D. catenatum* (64.51%) (Supplementary Figure S2). In addition, Class I (retrotransposons) and Class II (DNA transposons) TEs account for 49.96% and 8.42% of the *G. elata* genome, respectively (Figure 3B; Table 4). The quality of identified repetitive elements in these orchid species was assessed using LAI value (Supplementary Table S4). Although the LAI value in *G. elata* is slightly lower than *A. shenzhenica* and *D. catenatum*, *G. elata* shows the highest content of TEs (Supplementary Table S4). The LAI value for *P. equestris* could not be calculated as the proportion of intact TE was less than 0.05%. Like other sequenced orchid genomes, LTR retrotransposons, mainly Gypsy-type and Copia-type LTRs, are predominant (49.95%), followed in frequency by long interspersed nuclear elements (LINEs), which account for 4.33% of the genome. Of the repetitive elements, 15.32% could not be classified into any known families. In addition, the insertion time of LTR elements was estimated (Supplementary Figure S3), and the most abundant Gypsy-type LTRs were inserted relatively a long time ago and may have become fragmented and thus produce a lower LAI value. In summary, the repeat content of *G. elata*, especially LTR Gypsy elements, was larger than the other species in Orchidaceae family, which are not mycoheterotrophic.

**Table 3:** Statistics for *G. elata* genome annotation

Features	No. of features	Total length of features (bp)	**Average length** **of features (bp)**	Density (#/Mb)
Gene	18,698	133,969,721	7,164.92	17.87
CDS	18,844	17,679,423	938.20	18.01
Exon	88,096	25,125,034	285.20	84.21
Intron	69,252	109,135,442	1,575.92	66.20
3′ UTR	12,708	4,516,303	355.39	12.15
5′ UTR	11,657	2,932,215	251.54	11.14

**Table 4:** Sequence percentage (%) of annotated TEs proportional to the entire genome of *G. elata* and three species in the Orchidaceae family

		*G. elata*	*A. shenzhenica*	*P. equestris*	*D. catenatum*
DNA transposon	DNA	4.09	6.50	3.17	3.20
	LINE^*a*^	4.33	4.99	4.37	7.04
Retrotransposon	SINE^*b*^	0.01	0.08	0.04	0.07
	LTR^*c*^	49.95	13.71	32.66	34.19
	Gypsy	38.92	7.35	27.00	11.34
	Copia	4.61	3.46	4.49	19.35
Other	Unknown	15.32	0.27	20.78	18.29

a LINE, long interspersed nuclear element.

b SINE, short interspersed nuclear element.

c LTR, long terminal repeat.

The LTR elements are known to be the main drivers of gene evolution (Galindo-González *et al.* 2017), and they could have contributed to the gene loss and formation of unique genes in *G. elata*.

### Gene annotation and comparative analysis

**Figure 4:**
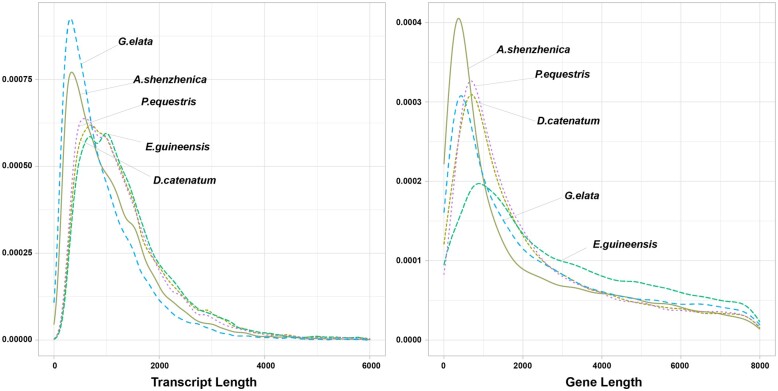
The distribution of transcript and gene length between *G. elata*, the other three species (*A. shenzhenica*, *D. catenatu*, and *P. equestris*) in Orchidaceae, and *E. guineensis*.

The complete annotated *G. elata* genome contains a final gene set comprising 18,844 CDS, with an AED less than 0.5 (Table 3). These CDSs total 17.68 Mb, and there is an average of 4.711 exons per gene. Among the final gene set, 15,619 CDSs are annotated in more than one database, including Uniprot, InterPro, Pfam, GO, and KEGG (Supplementary Table S5). The GO term analysis of the predicted proteome identified a number of proteins involved in metabolic and cellular processes, catalytic and binding activity, and cellular anatomical entity (Supplementary Figure S4). To compare gene content in *G. elata* and related species, we analyzed CDS distribution and gene length in *G. elata* relative to three other species in the Orchidaceae family (*A. shenzhenica, D. catenatum*, and *P. equestris*) and *Elaeis guineensis* (oil palm), as an outgroup (Figure 4). We found that *G. elata* shows the highest frequency of shorter length transcripts relative to other species. However, the overall gene-length distribution of *G. elata* is similar to that of other Orchidaceae species, except *A. shenzhenica*, which contains a genome that is smaller than the other species in this family (Figure 4; Supplementary Table S6). Last, the number of rRNAs and tRNAs predicted were 439 and 940, respectively.

### Orthology and gene family contraction and expansion

**Figure 5:**
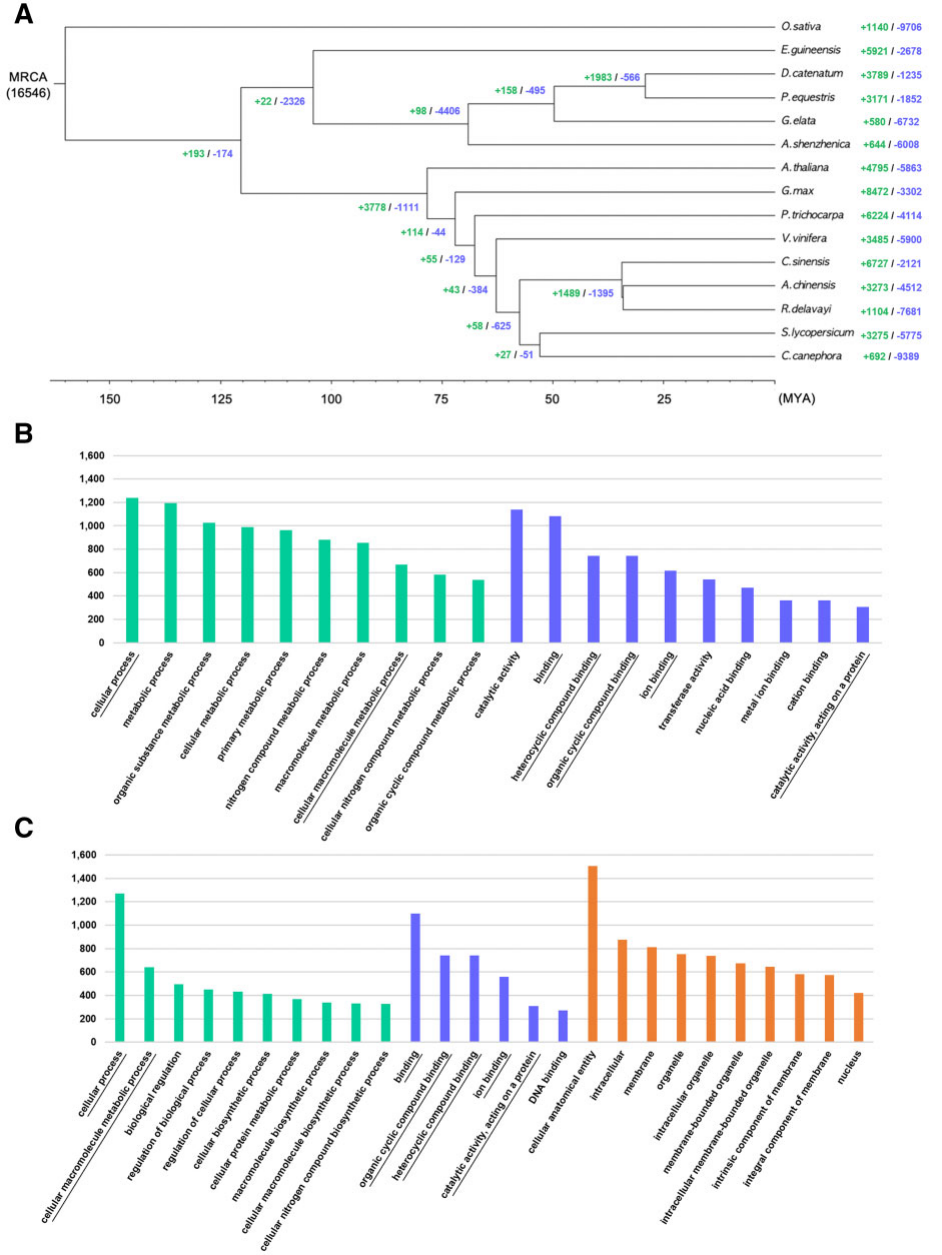
(A) Phylogenetic analysis of *G. elata* among 15 plants and gene family gain-and-loss analysis including the number of gained gene families (+) and lost gene families (−). (B) The number of genes in the top 10 GO terms of expanded gene families (Supplementary Table S7) and (C) contracted gene families (Supplementary Table S8) in the *G. elata* genome. The green, blue, and orange colored bars represent the three major GO categories, biological process, MF, and CE, respectively. The overlapping terms in both expanded and contracted gene families are underlined.

We next constructed a phylogenetic tree with *G. elata* and 14 other plants (Figure 5A). *G.elata* was found to cluster with other members of the Orchidaceae family, including *A. shenzhenica*, *D. catenatum*, and *P. equestris*. The tree shows that the Epidendroideae subfamily, which includes *G. elata, D. catenatum*, and *P. equestris* diverged from the Apostasioideae subfamily, which includes *A. shenzhenica*, approximately 65–70 million years ago (Mya). Gene family expansion and contraction analysis showed that substantial contraction occurred throughout divergence within the Orchidaceae family (Figure 5A). Notably, *A. shenzhenica* experienced gene loss due to a whole-genome duplication event, as previously reported (Zhang *et al.*, 2017). *G.elata* also experienced extensive gene loss, whereas *P. equestris* and *D. catenatum* gained more genes than were lost through evolution. The *G. elata* genome, specifically, gained 580 gene families but lost 6732 gene families. We then performed GO term analyses of the expanded and contracted gene families in *G. elata* to assign putative functions (Figure 5, B and C). In the biological process (BP) category, genes related to metabolic process (GO:0008151) and those involved in the metabolism of macromolecules (GO:0043170), such as organic substances (GO:0071704) and nitrogen compounds (GO:0006807), were expanded (Figure 5B; Supplementary Table S7). Although not appearing in the top 50 most frequently identified GO terms (Supplementary Table S7), genes involved in arbuscular mycorrhizal association (GO:00036277) were detected. In the molecular function (MF) category, genes involved in catalytic activity (GO:0003824) and transferase activity (GO:0016740) were expanded (Figure 5B; Supplementary Table S7). Conversely, significantly contracted genes include those related to biosynthetic and metabolic processes in BP, DNA binding (GO:0003677) in MF, and genes in the cellular component (CE) category (Figure 5C; Supplementary Table S8). The loss of genes involved in biosynthetic processes and CE reflects that such features of *G. elata* may depend on its symbiont partners. In addition, the expansion of genes with novel metabolic processes and binding activities may be rewiring due to the lifestyle transition of *G. elata* to fully mycoheterotrophic.

**Figure 6:**
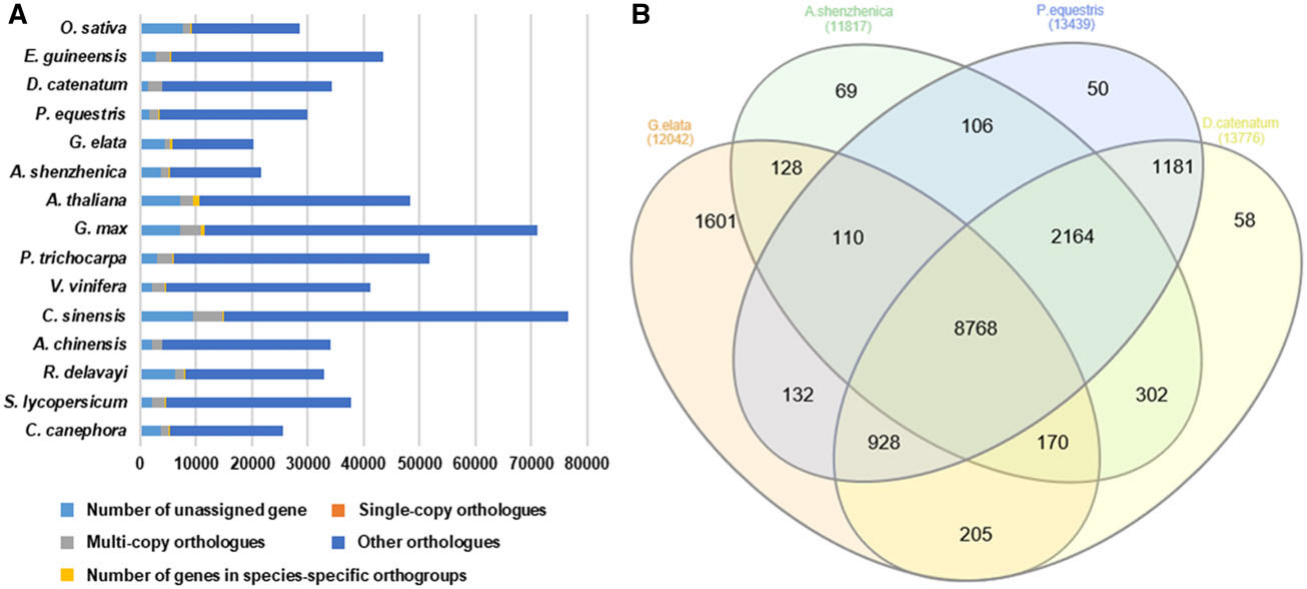
(A) Bar graph of the number of protein-coding genes in the 15 plant species including *G. elata*. The distribution of number of genes between *G. elata* and other 14 species by the type of orthogroups. Single-copy orthologs include common orthologs with one copy in all species. Multi-copy orthologs include common orthologs with multiple copy numbers in all species. The number of genes in species-specific orthogroups represents unique genes in specific species. Other orthologs include gene from families shared in 2–14 species. (B) Venn diagram of orthologous gene families between *G. elata* and other three species (*A. shenzhenica, D. catenatu*, and *P. equestris*) in the Orchidaceae family.

Orthology analysis with the 15 plant species included in our phylogenetic tree identified 16,115 orthologous gene families and 418 species-specific gene families (Supplementary Table S9). *G.elata* contains the lowest number of protein-coding genes compared to the other plant species and even to the other orchid species (Figure 6A; Supplementary Table S6). Conversely, of all the orchid species, *G. elata* encodes the highest number of unassigned genes and genes in species-specific orthogroups. We further identified a set of orthologous gene families shared among the orchid species (Figure 6B). This set contains a total of 8768 orthogroups that are conserved across all four orchid genomes, with an additional 928 orthogroups conserved across the three species in the Epidendroideae subfamily (*i.e.*, *G. elata*, *P. equestris*, and *D. catenatum*). *G.elata* encodes 1601 species-specific orthologs, which is more than the other Epidendroideae species.

We found that the genome of *G. elata* has an extremely low gene density, proliferation of repeat content, and significant expansion and contraction of genes involved in metabolic processes and biosynthetic processes, respectively. In addition, *G. elata* has the highest number of unique genes among the compared orchid species. Since nutrient absorption of this obligate mycoheterotrophic plant is entirely dependent on their fungal partners (Merckx *et al.* 2009), these genomic features may reflect the mycoheterotrophic and symbiotic lifestyle of the *G. elata*.

## Conclusion

Here, we report the first high-quality chromosome-level genome assembly of *G. elata*. We found an extremely low gene density, proliferation of repeat content, and significant contraction of genes involved in CEs, reflecting on its mycoheterotrophic lifestyle. Consequently, this high-quality reference genome data of *G. elata* will be important for informing further studies aimed at better understanding genomic interactions and gene expression changes that occur during the development of *G. elata* with its associated fungi, thereby uncovering the symbiotic mysteries underlying such mycoheterotrophic lifestyles.

## Data availability

The *G. elata* genome project was deposited at NCBI, under BioProject No. PRJNA632604. The raw DNA sequencing reads are available at the Sequence Read Archive (SRA) under Accession Nos. SRR12263394, SRR12263395, and SRR12263396. The raw Iso-Seq data is available under the Accession No. SRR13516450. The genome assembly data have been deposited at GenBank under the Accession No. GCA_016760335.1 (WGS: JACERR000000000.1). The commands and parameters used for the genome assembly and repeat annotation are available in Supplementary Table S10.

Supplementary material is available at *G3* online.

## Supplementary Material

jkab433_Supplemental_Tables

jkab433_Supplemental_Figures
