# Supplementary material for: Chromosome-level genome assembly of the fully mycoheterotrophic orchid Gastrodia elata
Source: G3 (Bethesda). 2022 Jan 19;12(3):jkab433. doi: 10.1093/g3journal/jkab433 (PMC8896018; doi:10.1093/g3journal/jkab433)
Supplement: jkab433_Supplemental_Tables [file jkab433_supplemental_tables.pdf]

Table S1. Genome assemblies used in this study.

Common name	Species	Accession Number	Reference
Kiwifruit	*Actinidia chinensis*	GCA_003024255.1	Pilkington *et al*., 2018
Grass orchids	*Apostasia shenzhenica*	GCA_002786265.1	Zhang *et al*., 2017
Thale cress	*Arabidopsis thaliana*	TAIR10	The Arabidopsis Genome Initiative. 2000
Robusta coffee	*Coffea canephora*	GCA_900059795.1	Denoeud *et al*., 2015
Tea plant	*Camellia sinensis*	GCA_004153795.2	Wei *et al*., 2018
Chained Dendrobium	*Dendrobium catenatum*	GCF_001605985.2	Zhang *et al*., 2016
African oil palm	*Elaeis guineensis*	GCF_000442705.1	Singh *et al*., 2013
Tien Ma	*Gastrodia elata*	JACERR000000000	This Study
Soybean	*Glycine max*	GCF_000004515.5	Schmutz *et al*., 2010
Rice	*Oryza sativa*	IRGSP-1.0	Goff *et al*., 2002
Horse Phalaenopsis	*Phalaenopsis equestris*	GCF_001263595.1	Cai *et al*., 2015
Black cottonwood	*Populus trichocarpa*	GCF_000002775.4	Tuskan *et al*., 2006
Rhododendron	*Rhododendron delavayi*	DOI: 10.5524/100331	Zhang *et al*., 2017
Tomato	*Solanum lycopersicum*	GCF_000188115.4	Hosmani *et al*., 2018
Grape	*Vitis vinifera*	GCF_000003745.3	Jaillon *et al*., 2007

Table S2. Sequencing data used *G. elata* genome assembly

	Platform	Insert size	# of Bases	Sequencing coverage (x)
DNA	PacBio Sequel	20 Kb	88,406,384,499	84.60
	Illumina Novaseq 6000	350 bp	132,112,232,346	126.43
	Hi-C	-	27,810,468,186	27.57
RNA	PacBio Sequel	1~6 Kb	72,377,817,589	69.26

Table S3. Lengths of *G. elata* genome assembly

Scaffolds	Length(bp)	% of Total Bases
Scx7bQ7_8	130,552,502	12.49
Scx7bQ7_10	105,251,433	10.07
Scx7bQ7_4	73,478,629	7.03
Scx7bQ7_1	69,223,480	6.62
Scx7bQ7_5	62,300,322	5.96
Scx7bQ7_14	52,263,389	5.00
Scx7bQ7_12	50,595,616	4.84
Scx7bQ7_3	50,555,507	4.84
Scx7bQ7_17	49,986,487	4.78
Scx7bQ7_7	48,229,918	4.62
Scx7bQ7_6	45,687,624	4.37
Scx7bQ7_19	44,908,175	4.30
Scx7bQ7_13	43,882,508	4.20
Scx7bQ7_11	40,290,820	3.86
Scx7bQ7_2	39,714,827	3.80
Scx7bQ7_9	36,014,257	3.45
Scx7bQ7_18	33,358,019	3.19
Scx7bQ7_16	32,099,965	3.07
Total	1,008,393,478	96.50

Table S4. Quality assessment of repetitive elements identified in four orchid species

Species	Intact	Total	Raw LAI	LAI
G. elata	0.0082	0.6227	1.31	6.94
A. shenzhenica	0.0037	0.1967	1.9	7.53
D. catenatum	0.0075	0.387	1.93	7.56
P. equestris	<0.0005	-	-	-

LAI: LTR Assembly Index

Table S5. Search for database search with the *G. elata* annotations

Database	# of mRNAs	% via Total mRNAs
Uni-prot/swiss-prot	18,545	98.41
InterProScan	14,772	78.39
Pfam	11,503	61.04
GO	15,619	82.89
KEGG	2,499	13.26

Table S6. Statistics of annotations for five species.

Species/Features	Number of genes	Total length of genes (bp)	Average length of genes (bp)	Percentage of total length of genes (%)	Number of CDSs	Total length of CDSs (bp)	Average length of CDSs (bp)	Percentage of total length of CDSs (%)	Density (#/Mb)
A.shenzhenica	21,743	133,669,749	6,148	38.33	21,743	23,901,705	1,099	6.85	62.35
D.catenatum	25,251	279,077,958	11,052	25.27	34,389	47,770,530	1,389	4.33	22.87
E.guineensis	29,569	249,417,635	8,435	16.25	43,551	62,228,253	1,429	4.05	19.26
G.elata	18,698	133,969,721	7,165	12.80	18,844	17,679,423	938	1.69	18.01
P.equestris	21,219	309,851,873	14,603	29.12	29,894	39,623,205	1,325	3.72	19.94

Table S7. Top 50 GO terms of expanded gene families in the *G. elata* genome among fifteen plants.

Category	GO ID	GO Name	No. genes	FDR
B	GO:0009987	cellular process	1,240	2.E-02
B	GO:0008152	metabolic process	1,195	3.E-06
M	GO:0003824	catalytic activity	1,139	2.E-04
M	GO:0005488	binding	1,082	2.E-10
B	GO:0071704	organic substance metabolic process	1,026	2.E-05
B	GO:0044237	cellular metabolic process	990	2.E-05
B	GO:0044238	primary metabolic process	964	5.E-04
B	GO:0006807	nitrogen compound metabolic process	882	2.E-05
B	GO:0043170	macromolecule metabolic process	856	4.E-08
M	GO:1901363	heterocyclic compound binding	744	2.E-05
M	GO:0097159	organic cyclic compound binding	744	3.E-05
B	GO:0044260	cellular macromolecule metabolic process	667	1.E-09
M	GO:0043167	ion binding	617	6.E-09
B	GO:0034641	cellular nitrogen compound metabolic process	582	2.E-04
M	GO:0016740	transferase activity	541	2.E-05
B	GO:1901360	organic cyclic compound metabolic process	536	8.E-05
B	GO:0006725	cellular aromatic compound metabolic process	531	3.E-05
B	GO:0046483	heterocycle metabolic process	526	4.E-05
B	GO:0006139	nucleobase-containing compound metabolic process	506	3.E-05
B	GO:0090304	nucleic acid metabolic process	485	2.E-07
M	GO:0003676	nucleic acid binding	470	3.E-06
B	GO:0009058	biosynthetic process	434	1.E-02
B	GO:1901576	organic substance biosynthetic process	423	2.E-02
B	GO:0044249	cellular biosynthetic process	411	3.E-02
B	GO:0019538	protein metabolic process	410	5.E-02
B	GO:0065007	biological regulation	394	2.E-02
B	GO:0050789	regulation of biological process	366	2.E-03
M	GO:0046872	metal ion binding	363	8.E-12
M	GO:0043169	cation binding	363	4.E-11
B	GO:0010467	gene expression	360	4.E-04
B	GO:0016070	RNA metabolic process	357	2.E-02
B	GO:0009059	macromolecule biosynthetic process	334	1.E-03
B	GO:0044271	cellular nitrogen compound biosynthetic process	331	6.E-04
B	GO:0034645	cellular macromolecule biosynthetic process	329	1.E-03
B	GO:0043412	macromolecule modification	313	3.E-02

M	GO:0140096	catalytic activity, acting on a protein	308	6.E-03
B	GO:1901362	organic cyclic compound biosynthetic process	280	2.E-05
B	GO:0018130	heterocycle biosynthetic process	277	2.E-06
B	GO:0019438	aromatic compound biosynthetic process	272	1.E-05
B	GO:0019222	regulation of metabolic process	264	1.E-05
B	GO:0034654	nucleobase-containing compound biosynthetic process	260	7.E-07
B	GO:0060255	regulation of macromolecule metabolic process	257	1.E-05
M	GO:0046914	transition metal ion binding	231	8.E-21
B	GO:0031323	regulation of cellular metabolic process	223	4.E-03
B	GO:0010468	regulation of gene expression	221	2.E-05
B	GO:0080090	regulation of primary metabolic process	218	6.E-03
B	GO:0051171	regulation of nitrogen compound metabolic process	216	7.E-03
M	GO:0008270	zinc ion binding	199	4.E-26
B	GO:0009889	regulation of biosynthetic process	189	2.E-03
B	GO:0031326	regulation of cellular biosynthetic process	188	3.E-03

Table S8. Top 50 GO terms of contracted gene families in the *G. elata* genome among fifteen plants.

Category	GO ID	GO Name	No. genes	FDR
C	GO:0110165	cellular anatomical entity	1,505	7.E-15
B	GO:0009987	cellular process	1,270	1.E-03
M	GO:0005488	binding	1,098	2.E-11
C	GO:0005622	intracellular	877	7.E-03
C	GO:0016020	membrane	813	5.E-05
C	GO:0043226	organelle	753	1.E-03
M	GO:0097159	organic cyclic compound binding	741	2.E-04
M	GO:1901363	heterocyclic compound binding	740	2.E-04
C	GO:0043229	intracellular organelle	739	2.E-03
C	GO:0043227	membrane-bounded organelle	674	9.E-03
C	GO:0043231	intracellular membrane-bounded organelle	645	3.E-02
B	GO:0044260	cellular macromolecule metabolic process	642	9.E-06
C	GO:0031224	intrinsic component of membrane	580	2.E-02
C	GO:0016021	integral component of membrane	572	2.E-02
M	GO:0043167	ion binding	558	2.E-02
B	GO:0065007	biological regulation	496	1.E-16
B	GO:0050789	regulation of biological process	452	5.E-17
B	GO:0050794	regulation of cellular process	432	2.E-19
C	GO:0005634	nucleus	420	5.E-10
B	GO:0044249	cellular biosynthetic process	414	3.E-02
B	GO:0044267	cellular protein metabolic process	370	9.E-03
B	GO:0009059	macromolecule biosynthetic process	337	9.E-04
B	GO:0034645	cellular macromolecule biosynthetic process	331	9.E-04
B	GO:0044271	cellular nitrogen compound biosynthetic process	328	2.E-03
M	GO:0140096	catalytic activity, acting on a protein	308	8.E-03
B	GO:0006793	phosphorus metabolic process	298	6.E-05
B	GO:0006796	phosphate-containing compound metabolic process	297	4.E-05
B	GO:0019222	regulation of metabolic process	296	1.E-11
B	GO:0050896	response to stimulus	294	3.E-07
B	GO:0060255	regulation of macromolecule metabolic process	291	4.E-12
B	GO:0006464	cellular protein modification process	283	9.E-05
B	GO:0036211	protein modification process	283	9.E-05
B	GO:0031323	regulation of cellular metabolic process	282	8.E-14
B	GO:1901362	organic cyclic compound biosynthetic process	280	4.E-05
B	GO:0051179	localization	278	5.E-04

B	GO:0080090	regulation of primary metabolic process	277	9.E-14
B	GO:0051171	regulation of nitrogen compound metabolic process	276	8.E-14
B	GO:0019438	aromatic compound biosynthetic process	272	2.E-05
M	GO:0003677	DNA binding	270	3.E-14
B	GO:0018130	heterocycle biosynthetic process	265	2.E-04
B	GO:0051234	establishment of localization	265	2.E-03
B	GO:0006810	transport	264	8.E-04
B	GO:0034654	nucleobase-containing compound biosynthetic process	258	3.E-06
B	GO:0010468	regulation of gene expression	248	1.E-10
B	GO:0009889	regulation of biosynthetic process	238	3.E-13
B	GO:0019219	regulation of nucleobase-containing compound metabolic process	237	2.E-13
B	GO:0031326	regulation of cellular biosynthetic process	237	4.E-13
B	GO:0010556	regulation of macromolecule biosynthetic process	236	2.E-13
B	GO:0032774	RNA biosynthetic process	236	2.E-12
B	GO:0051252	regulation of RNA metabolic process	234	9.E-14

Table S9. Statistics of gene families of fifteen plants.

	Statistics
Number of species	15
Number of genes	439,045
Number of genes in orthogroups	387,848
Number of unassigned genes	51,197
Percentage of genes in orthogroups	88.3
Percentage of unassigned genes	11.7
Number of orthogroups	16,115
Number of species-specific orthogroups	418
Number of genes in species-specific orthogroups	2,718
Percentage of genes in species-specific orthogroups	0.6
Mean orthogroup size	24.1
Median orthogroup size	18
G50 (assigned genes)	31
G50 (all genes)	27
O50 (assigned genes)	3,194
O50 (all genes)	4,091
Number of orthogroups with all species present	6,642
Number of single-copy orthogroups	123

Table S10. The program commands and parameters for genome assembly and repeat annotation of *G. elata*.

Process	Program	Parameter
Kmer Analysis	jellyfish	jellyfish count -F 2 -m 17 -C -s 10G -t 32
Assembly	FALCON-Unzip	length_cutoff=13000<br>pa_HPCdaligner_option=-v -B128 -M24<br>pa_daligner_option= -k18 -e0.75 -l1200 -h256 -w8 -s100<br>falcon_sense_option=--output-multi --min-idt 0.70 --min-cov 4 --max-n-read 200<br>length_cutoff_pr = 10000<br>overlap_filtering_setting=--max-diff 60 --max-cov 60 --min-cov 2
Hi-C scaffolding	HiRise	(Not provided from Dovetail Genomics)
Repeat Analysis	RepeatModeler	BuildDatabase -engine ncbi<br>RepeatModeler -engine ncbi -pa 24
	LTR_retrever	gt suffixerator -tis -suf -lcp -des -ssp -sds -dna<br>gt ltrharvest -minlenltr 100 -maxlenltr 7000 -mintsd 4 -maxtsd 6 -motif TGCA -motifmis 1 -similar 85 -vic 10 -seed 20 -seqids yes<br>LTR_FINDER_parallel -threads 10 -harvest_out -size 1000000 -time 300<br>LTR_retriever -threads 10
	RepeatMasker	RepeatMasker -lib -pa 32 -no_is -dir
