# Supplementary material for: Chromosome-level genome assembly of the fully mycoheterotrophic orchid Gastrodia elata
Source: G3 (Bethesda). 2022 Jan 19;12(3):jkab433. doi: 10.1093/g3journal/jkab433 (PMC8896018; doi:10.1093/g3journal/jkab433)
Supplement: jkab433_Supplemental_Figures [file jkab433_supplemental_figures.pdf]

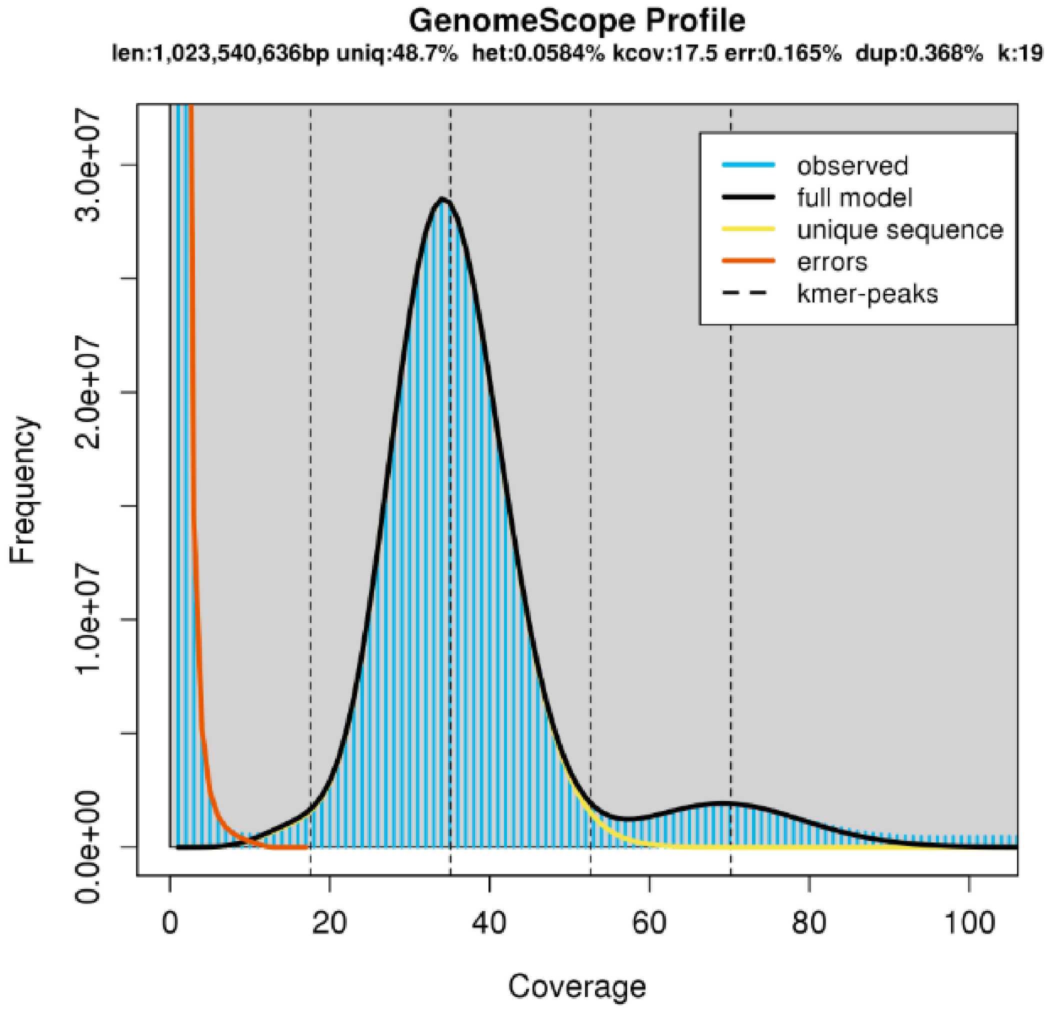

Figure S1. Genome characteristics information of *G. elata* using GenomeScope.

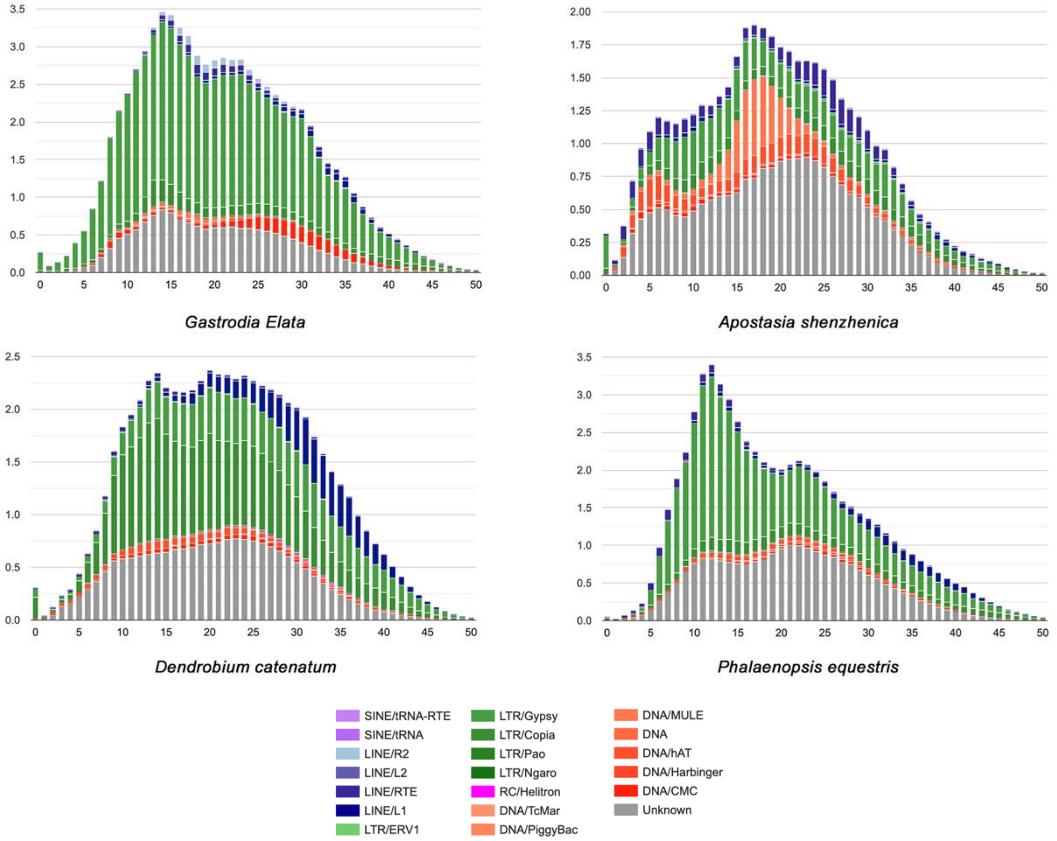

Figure S2. Kimura distance-based copy divergence analysis of TEs in the Orchidaceae family genomes (*G. elata*, *A. shenzhenica*, *D. catanatum* and *P. equwstris*). The graphs represent genome coverage (y-axis) for each type of TEs (DNA transposons, SINE, LINE, and LTR retrotransposons) in Kimura distances to their corresponding consensus sequence (x axis, K-value from 0 to 50).

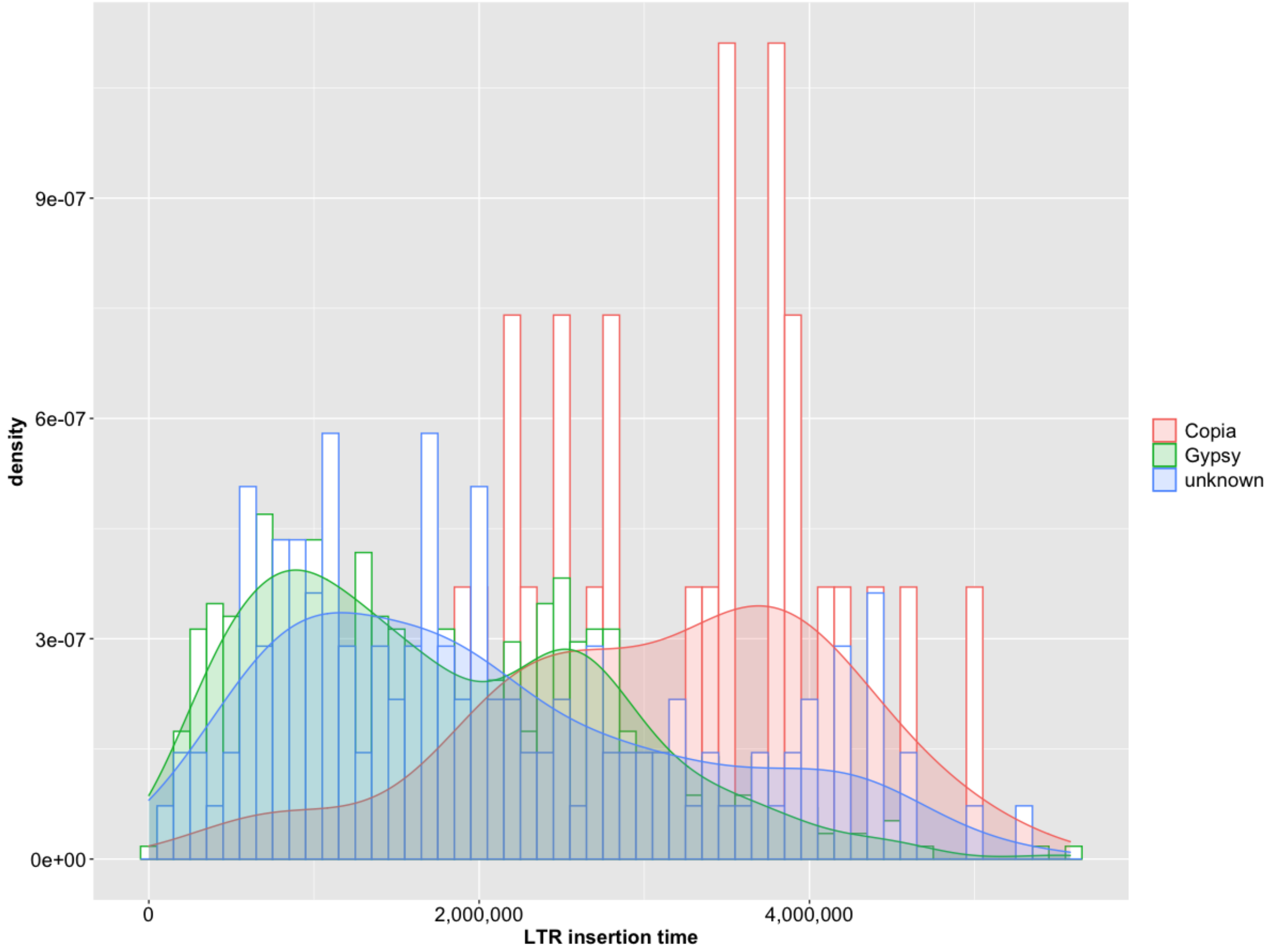

Figure S3. Estimated insertion time (mya) of LTRs in *G. elata* and the density of elements at each insertion time.

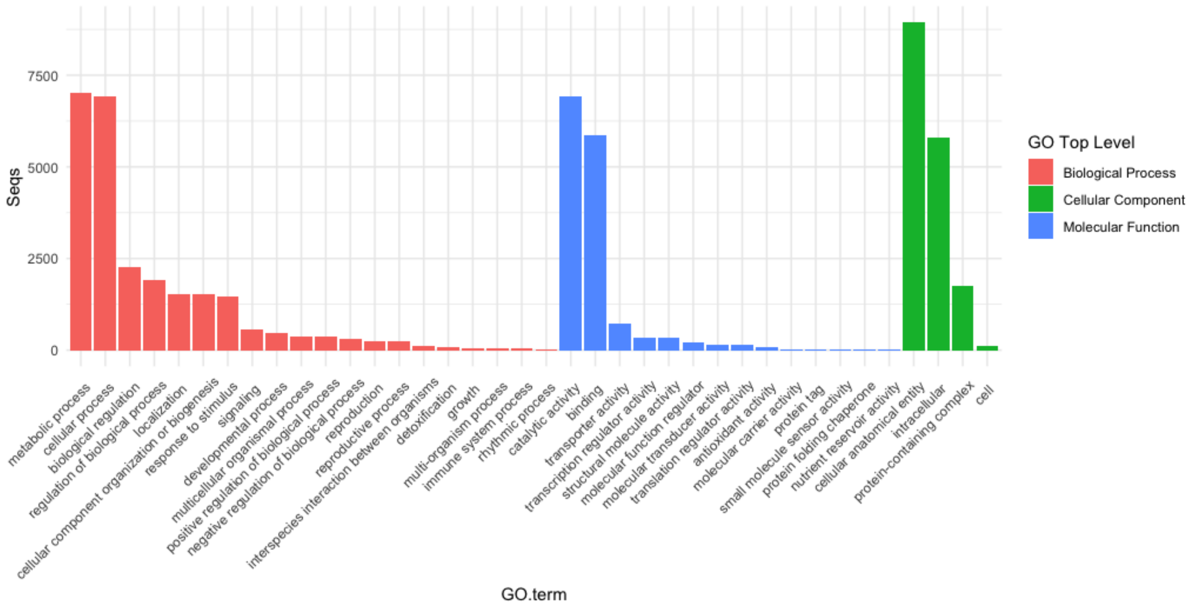

Figure S4. GO annotation of the predicted genes of the *G. elata* genome. The horizontal axis indicates classes of the second-level GO-annotation, and the vertical level axis indicated the number of genes in each class.
